# Artificial intelligence enhanced ophthalmological screening in children: insights from a cohort study in Lubelskie Voivodeship

**DOI:** 10.1038/s41598-023-50665-5

**Published:** 2024-01-02

**Authors:** Regulski Piotr, Rejdak Robert, Niezgódka Marek, Iwański Michał

**Affiliations:** 1 Laboratory of Digital Imaging and Virtual Reality, Department of Dental and Maxillofacial Radiology, Medical University of Warsaw, Binieckiego 6 St., 02-097 Warsaw, Poland; 2 Chair and Department of General and Pediatric Ophthalmology, Medical University of Lublin, Lublin, Poland; 3 Silesian University of Technology, Gliwice, Poland

**Keywords:** Epidemiology, Paediatric research

## Abstract

This study aims to investigate the prevalence of visual impairments, such as myopia, hyperopia, and astigmatism, among school-age children (7–9 years) in Lubelskie Voivodeship (Republic of Poland) and apply artificial intelligence (AI) in the detection of severe ocular diseases. A total of 1049 participants (1.7% of the total child population in the region) were examined through a combination of standardized visual acuity tests, autorefraction, and assessment of fundus images by a convolutional neural network (CNN) model. The results from this artificial intelligence (AI) model were juxtaposed with assessments conducted by two experienced ophthalmologists to gauge the model's accuracy. The results demonstrated myopia, hyperopia, and astigmatism prevalences of 3.7%, 16.9%, and 7.8%, respectively, with myopia showing a significant age-related increase and hyperopia decreasing with age. The AI model performance was evaluated using the Dice coefficient, reaching 93.3%, indicating that the CNN model was highly accurate. The study underscores the utility of AI in the early detection and diagnosis of severe ocular diseases, providing a foundation for future research to improve paediatric ophthalmic screening and treatment outcomes.

## Introduction

The increasing prevalence of visual impairments in children, particularly myopia, hyperopia, and astigmatism, has become a significant global health concern^1–3^. Not only can these conditions impede academic achievement, but they may also, in severe cases, lead to blindness, underscoring the urgency of early intervention^4–6^. Timely detection and proper management are crucial in mitigating these risks and ensuring the overall well-being of children^7–9^. Early intervention, predicated upon prompt and accurate diagnosis in pediatric cases, is imperative for the efficacious implementation of appropriate therapeutic measures.

In recent times, the COVID-19 pandemic has further complicated this scenario. The impact of the outbreak per se and its social consequences proved devastating in many respects for children^10,11^. Long-lasting distance learning and reduced physical activities have all contributed to the development of significant negative effects on the health of children, particularly vision impairment and eye disorders. This alarming trend underscored the need for urgent and effective health measures, including early detection and appropriate preventive strategies. Massive screening diagnostic actions should be undertaken to diagnose ocular diseases early and prevent their development in children. Screening programs should be undertaken utilizing advanced diagnostic technology (including artificial intelligence—AI), which will enable a noninvasive approach and require limited technical staff involvement.

However, the application of AI in the diagnostic screening programs of visual disorders among children is rarely documented in the literature^12^. Notwithstanding, the current study focuses on overcoming this disparity in through the use of AI-driven approach. In particular, a convolutional neural network (CNN) algorithm is deployed for automatic analysis of images captured through a fundus camera. In addition to identifying common visual impairments, the study further seeks to detect severe ocular diseases such as glaucoma, diabetic macular oedema, and macular degeneration.

The study herein focuses on investigating the prevalence of these visual impairments in school-age children. This screening, prospective, cohort study aims to enhance our understanding of these common visual conditions and explores the application of innovative technologies to improve the detection and management of severe ocular diseases. The purpose of the study was to assess the prevalence of visual impairments, namely, myopia, hyperopia, and astigmatism, in school-age children aged 7–9 years from the Lublin Voivodeship. Furthermore, for children diagnosed with these visual impairments, the study aimed to identify the presence or absence of severe eye diseases, including glaucoma, diabetic macular oedema, and macular degeneration, using fundus camera images and automatic analysis via an AI algorithm based on a convolutional neural networks (CNN). The study also intended to contrast the accuracy of the AI algorithm's assessments with those conducted by two medical doctors specializing in ophthalmology. The expected outcome of this study is validation of the accuracy and efficiency of AI algorithms in diagnosing severe eye diseases in children, with the potential for such technology to supplement or enhance traditional diagnostic methods. This could lead to earlier and more accurate detection of severe eye diseases, improving treatment outcomes and quality of life for affected children.

## Results

The ophthalmic screening study encompassed children aged 7–9 years who were attending grades 1–3 in primary schools. Ten participating schools were randomly chosen to mirror the demographic structure of the voivodeship. Of these, five were situated in rural regions, and the remainder were located in urban areas. This selection methodology ensured an accurate representation of the child population within the voivodeship. Based on the final results of the 2021 National Census, the Lubelskie Voivodeship, as of March 31, 2021, had a population of 2,052,340 individuals, of whom 51.6% were females and 48.4% were males^13^. The population of children aged 7–9 years was reported to be 62,784. Therefore, in this cohort study, a representative 1.7% of the total population of children aged 7–9 years from the entire voivodeship was included^13^.

The study assessed a total of 1058 participants. However, two children were excluded due to challenges encountered during the examination, and an additional seven were omitted due to missing sex data in the study protocol. Consequently, the final dataset for subsequent analysis included 1049 children, with a nearly balanced gender distribution comprising 533 males and 516 females. Of the participants, 360 males and 332 females were from urban areas, while 173 males and 184 females originated from rural settings. In the first class, encompassing children approximately 7 years old (± 6 months), there were 177 males (111 urban, 66 rural) and 185 females (112 urban, 73 rural). The second class, with children around 8 years old (± 6 months), consisted of 187 male (125 urban, 62 rural) and 173 female (112 urban, 61 rural) participants. The second class, with children around 8 years old (± 6 months), consisted of 187 male (125 urban, 62 rural) and 173 female (112 urban, 61 rural) participants. In the study, individuals residing in the rural areas were characterized by a lower socioeconomic status, reflecting the socioeconomic disparities often observed between urban and rural populations. Comprehensive characteristics of the study population are delineated in Table 1.

**Table 1:** Characteristics of the study population.

Sex	School location	Class			Total
		1st (7 yo ± 6 mo)	2nd (8 yo ± 6 mo)	3rd (9 yo ± 6 mo)	
Male	Total	177	187	169	533
	Urban	111	125	124	360
	Rural	66	62	45	173
Female	Total	185	173	158	516
	Urban	112	112	108	332
	Rural	73	61	50	184
Total		362	360	327	1049

The participants underwent examination to evaluate their visual acuity using a Snellen chart. An autorefractor (Welch Allyn, Baxter International, NY, USA) was employed to measure various parameters, including the spherical power (SPH), spherical equivalent refraction (SER), cylindrical power (CYL), axis (α), and the J0 and J45 indices. Myopia was characterized for children presenting with an SPH ≤ − 0.75 dioptres (D) in at least one eye. Emmetropia was defined within the range of − 0.75 D ≤ SPH <  + 1 D, and hyperopia was attributed to cases where the SPH ≥  + 1 D. Astigmatism was diagnosed in instances where CYL ≤ − 1 D or CYL ≥  + 1 D^14^.

The observed prevalence of myopia, hyperopia, and astigmatism among the children was 3.7%, 16.9%, and 7.8%, respectively. Notably, the prevalence of myopia exhibited a significant age-related increase, escalating from 1.9% among first-grade students to 5.8% among third-grade students (χ^2^ = 7.2306, p = 0.0269). Conversely, the prevalence of hyperopia decreased significantly with age, declining from 21.8% in first-grade students to 11.9% in third-grade students (χ^2^ = 12.0886, p = 0.0024). The prevalence of astigmatism was significantly higher in urban areas (8.9%) than in rural schools (5.1%) (χ^2^ = 5.8346, p = 0.0236). However, there were no significant correlations between sex, age, and school region and eyeglass usage. Detailed results are enumerated in Table 2.

**Table 2:** Results of the clinical examination.

	Sex			Class				Location		
	Male	Female	p	1st	2nd	3rd	p	Urban	Rural	p
Clinical examination										
Myopia	21 (3.9%)	18 (3.5%)	0.7236	*7 (1.9%)*	*13 (3.6%)*	*19 (5.8%)*	***0.0269***	25 (3.6%)	14 (3.9%)	0.8025
Hyperopia	86 (16.1%)	91 (17.6%)	0.3045	*79 (21.8%)*	*59 (16.4%)*	*39 (11.9%)*	***0.0024***	126 (18.2%)	51 (14.3%)	0.1321
Astigmatism	43 (8.1%)	37 (7.2%)	0.5816	33 (9.1%)	24 (6.7%)	23 (7.1%)	0.4161	62 (8.9%)	18 (5.1%)	**0.0236**
Eyeglasses	55 (11.0%)	57 (12.1%)	0.6161	33 (9.9%)	40 (11.8%)	39 (12.8%)	0.4910	81 (11.7%)	31 (11.0%)	0.2307

Significant values are in bold and italics.

Parents of the participants completed a questionnaire addressing potential visual impairments, strabismus, and amblyopia as observed by them, as well as any history of ophthalmological examinations undertaken by their children. Based on their responses, significant variations were observed in perceived visual acuity across different grades (χ^2^ = 8.0172, p = 0.0182). Parents more frequently reported deteriorating vision in older age groups. Noteworthy disparities were detected in reports of amblyopia and previous ophthalmological examinations between urban and rural regions (p = 0.0029 and p = 0.0006, respectively). In urban areas, instances of amblyopia and a history of ophthalmological examinations were reported more frequently than in rural areas. Detailed results are listed in Table 3.

**Table 3:** Results of the questionnaire.

	Sex			Class				Location		
	Male	Female	p	1st	2nd	3rd	p	Urban	Rural	p
Questionnaire										
Squint	3 (0.6%)	4 (0.8%)	0.6759	3 (0.9%)	1 (0.3%)	3 (0.9%)	0.5246	5 (0.7%)	2 (0.6%)	0.7559
Amblyopia	105 (20.4%)	102 (19.1%)	0.6535	72 (19.8%)	71 (19.7%)	64 (19.5%)	0.9895	**145 (22.1%)**	**35 (12.7%)**	**0.0029**
Bed eyesight	*51 (9.6%)*	*67 (12.9%)*	*0.0817*	*32 (8.8%)*	*36 (10.0%)*	*50 (15.3%)*	***0.0182***	75 (10.8%)	43 (12.4%)	0.5586
Prior examination	226 (42.4%)	218 (42.3%)	0.9514	141 (37.9%)	152 (44.4%)	151 (44.8%)	0.1455	**319 (46.1%)**	**125 (35.0%)**	**0.0006**

Significant values are in bold and italics.

The patients were referred to the University Ophthalmology Clinic for extended diagnostics when visual acuity (using the Snellen chart) was less than 1.0, when SPH was less than or equal to − 0.75 D or equal to or exceeded + 1.0 D, or when the absolute value of CYL exceeded 1.0 D (astigmatism).

In total, 109 participants were subsequently referred for further diagnostics. The examination procedure at the clinic included the following steps: the ophthalmologist first evaluated the patients' distance visual acuity using Snellen charts. Next, anterior eye segment assessment was performed via slit-lamp examination. Following the application of pupil-dilating drops (cyclopentolate solution), refraction was measured with a stationary autorefractometer (VX90, Visionix, USA). Finally, the fundus was examined using a slit lamp, and a fundus photograph was captured with an OPTOS California device (Nikon, Japan).

The diagnosis was rendered based on an assessment of the fundus images by two ophthalmologists. The interrater reliability was evaluated using Cohen's kappa coefficient, demonstrating an excellent level of agreement with κ = 1. There were no instances of disagreement between the two ophthalmologists in their evaluations of the fundus images; in total, 7 children were diagnosed with optic disc degeneration (drusen). Two one-sided Student’s t tests (TOST) were utilized to verify equivalence between the parameters assessed in schools and those assessed at the clinic. The results indicated a statistically significant equivalence (p < 0.0001, TOST), suggesting that the tests conducted in school settings were equivalent to those performed in a clinical environment.

The AI model predicted the presence of drusen and potential degeneration in seven cases. These predictions were subsequently corroborated by ophthalmologists during clinical examinations of all seven children. Therefore, the model was accurate in detecting macular degeneration. In one case, the model predicted diabetic retinopathy; however, it was not confirmed in clinical examination. In a broader context, serious pathologies were not revealed in either automatic or clinical examinations for 101 children. The Dice coefficient, a statistical validation metric demonstrating the predictive accuracy of the model, yielded a high value of 93.3%, attesting to the model's efficiency and reliability in detecting ocular pathologies.

## Discussion

This comprehensive ophthalmic screening study among children aged 7–9 years in Lubelskie Voivodeship provides several notable insights into the prevalence and characteristics of visual impairments in this population. The total sample size represented 1.7% of the total population of children in this age group across the region, provides a substantial dataset for analysis. This proportion was meticulously chosen to provide a substantial and representative dataset for robust analysis, balancing comprehensiveness with logistical feasibility. The selection of 1.7% of the population was based on statistical considerations to ensure that the sample size was large enough to offer reliable insights while being manageable in terms of resource allocation and study logistics. The study utilized a stratified sample selection strategy accounting for the demographic structure of the voivodeship as well as the geographical distribution of rural and urban schools. While this design enhances the generalizability of the findings, the true representation of the sample could be verified through a comparison of the sample demographic characteristics with the larger population data.

The study findings highlight the prevalence of myopia, hyperopia, and astigmatism among the participants. In evaluating these conditions our methodology diverged from the conventional use of spherical equivalent alone. The spherical equivalent, while commonly employed due to its simplicity, combining both spherical and cylindrical components into a single value, has notable limitations. It can result in different refractive states being represented as identical, thereby potentially oversimplifying the individual complexities of refractive errors^15^. Moreover, as emphasized in the work of Galvis et al., relying solely on the spherical equivalent may not be sufficient for accurately defining refractive errors, especially in research settings where detailed assessments are crucial^16^. Therefore, in our study, we incorporated an analysis of both spherical and cylindrical components. This comprehensive approach is particularly critical in pediatric populations, where the implications of astigmatic components on visual development and ocular health are significant.

According to the data from Hashemi et al.^1^, the estimated prevalence of myopia, hyperopia, and astigmatism in children is reported to be 11.7%, 4.6%, and 14.9%, respectively. In the Bhutan School Sight Survey^17^, a study encompassing 4,985 school children, the researchers discovered a prevalence rate of 6.64% for myopia, 2.17% for hyperopia, and 9.75% for astigmatism. In a comprehensive ophthalmic survey conducted by Jrbashyan et al.^18^, a sample of 5944 schoolchildren aged 6–15 years, screened exhibited a 9.7% prevalence of vision impairment in at least one eye and a 0.05% incidence of blindness in at least one eye. The study identified myopia as the most prevalent refractive error, affecting 60% of the students with vision impairment, followed by astigmatism at 33.7%, hyperopia at 29.5%, and strabismus at 3.8%. Regionally, the variation in the prevalence of myopia is notable, with Southeast Asia showing a prevalence at 4.9%, lower than that of the Western Pacific region, where it climbs to 18.2%. Similarly, for hyperopia, the estimated prevalence varies from a low of 2.2% in Southeast Asia to a high of 14.3% in the Americas. Astigmatism demonstrates a similar pattern, with an estimated prevalence ranging from 9.8% in Southeast Asia to 27.2% in the Americas^2,3,19–21^.

The prevalence of myopia and astigmatism in our study was lower than that observed in populations worldwide; however, the prevalence of hyperopia was higher. The observed age-related increase in myopia and decrease in hyperopia prevalence are consistent with trends observed in other paediatric populations worldwide^21^. This suggests that age might be a significant determinant of these conditions. Notably, the prevalence of astigmatism was significantly higher in urban areas, highlighting a potential urban‒rural disparity that warrants further investigation.

Carlton et al.^22^ provided comprehensive research on vision screening programs in 46 countries, revealing a large variability in study screening protocols. Despite the widespread availability of 'gold standard' visual acuity tests, they are often not used in many vision screening programs. Up to 40% of countries rely solely on Snellen tests, and 30% report variations in test usage based on factors such as region and clinician preference. The age at which screenings are performed and the tests used also differ widely, and data on the effectiveness of these screenings are often lacking or not centrally collected. None of the surveys used AI-based screening^22^. This serves to underscore the significance of our study, which deploys AI-based screening techniques for assessing paediatric myopia. In this context, the lifelong aggregation of risk factors, especially during critical phases of visual maturation, significantly shapes the trajectories of visual function and contributes to pronounced regional disparities in visual impairment. This insight accentuates the urgent need for a more uniform and scientifically rigorous approach to vision screening^23^.

Considering the aforementioned inconsistencies in traditional vision screening programs across various countries^22^, our research introduces a level of standardization and data-driven accuracy that is markedly absent in current practices. The integration of AI in vision screening allows more consistent, rapid, and scalable evaluations, potentially leading to earlier detection and intervention strategies. Moreover, our AI-powered approach is capable of centrally aggregating data, thereby providing robust metrics for evaluating the efficacy of vision screening programs. This marks a paradigm shift in how paediatric ocular health could be monitored globally, emphasizing the transformative potential of our study.

The use of AI for detecting visual impairments in children is scarcely represented in the current scientific literature, primarily due to the small number of extensive and age-specific screening protocols for this population^24^. This void in the literature highlights a pressing need to develop and test advanced diagnostic methods that are adapted to young populations^6,25,26^. The present study addresses this need by implementing a CNN algorithm, thereby presenting an innovative approach to paediatric ophthalmic screening. Despite the single instance of overprediction where the CNN model identified diabetic retinopathy that was not confirmed by ophthalmologists, this occurrence should not be disregarded. It could potentially indicate a prediabetic state or an early stage of the disease that has not yet manifested in noticeable ocular changes. Given that diabetic retinopathy is a known complication of diabetes, this case may underscore the predictive capabilities of the AI model and its potential usefulness in preventive medicine. Therefore, we suggest that this child be monitored more closely for signs of diabetes development.

Further research in the realm of pediatric ophthalmology necessitates the aggregation of an expansive and varied array of pediatric eye condition data. The primary objective of this endeavor is to increase the volume of a dataset that is both extensive in quantity and diverse in the pathologies it encompasses. This dataset will serve as the foundational training material for developing AI algorithms. The sophistication and efficacy of these algorithms are contingent on the breadth and diversity of the training data, which should include a wide spectrum of pediatric ocular pathologies. By training on such a comprehensive dataset, AI models can be refined to become more generalizable and precise, thereby enhancing their diagnostic accuracy and utility in diverse clinical scenarios. Efforts should also be directed towards the integration of these AI tools into routine pediatric ophthalmic practice. This integration involves not only the technical implementation of AI systems into existing clinical workflows but also a focus on ensuring that these tools are intuitive and user-friendly for ophthalmologists.

In conclusion, this study provides a comprehensive evaluation of ocular health among children in the Lublin voivodeship, underscoring the prevalence of common visual impairments and the potential role of factors such as age and geographical location. The findings also highlight the promise of innovative technologies, such as AI, in enhancing the diagnosis and management of paediatric ocular conditions. Future research should seek to replicate and extend these findings, exploring the underlying causes of the observed trends and examining the long-term impact of these conditions on children's educational and health outcomes.

## Methods

The research pertaining to ophthalmic screening encompassed children within the age range of 7–9 years who were attending grades 1–3 in primary education institutions in the Lubelskie Voivodeship (Poland). A total of ten schools, selected to mirror the demographic distribution of the voivodeship, were involved in the study. The geographical location of these schools was bifurcated such that five were situated in rural settings, while the rest were nested in urban environments^13^. The sample size for this study was determined using a power analysis. This was conducted with the specific aim of detecting differences in the incidence of myopia among different age groups. Based on an expected effect size, a significance level of 0.05, and a power of 0.80, the minimum required sample size was determined to be 1005^28,29^. To account for an estimated dropout rate of 5%, we initially recruited 1058 children for this study.

The research was carried out from September 2021 to May 2022. In September, the participating schools were randomly selected. From November to March, the children underwent ophthalmic examinations at their respective schools. Systematic vision screening was conducted in classroom environment to identify children with visual impairments, such as myopia, hyperopia, and astigmatism. Standardized visual acuity tests and refraction exams were performed by trained optometrists. The subjects participated in an examination to assess vision sharpness with the use of a Snellen chart at a test distance of 6 m within well-lit classrooms, optimizing conditions for accurate measurements. Trained optometrists administered the test, instructing the children to cover one eye at a time and read aloud the letters or symbols from top to bottom. Each line on the Snellen chart corresponded to a specific visual acuity level, allowing for a standardized measurement of each child's visual sharpness. The visual parameters, such as SPH, SER, CYL, axis (α), and the J0 and J45 indices, were measured utilizing an autorefractor from Welch Allyn for a subsequent examination involving the use of a refractometer and fundus examination with an OPTOS California Nikon device. The device’s ultra-wide-field imaging technology enabled us to capture more than 80% of the retina in a single image, providing a comprehensive view of the retinal health. Follow-up examinations for these children were conducted in April and May 2022. A total of 81% of the referred children reported to the clinic for their appointments.

The prevalence of myopia, hyperopia and astigmatism was calculated. Statistical analysis was performed to assess the relationship between these variables and the following parameters: sex, age, and urban/rural area. The chi-squared test was employed to compare the following nominal variables between groups: vision defects (myopia/emmeropia/hyperopia), astigmatism, wearing glasses. The questionnaire was instrumental in augmenting the clinical findings with parental observations and historical information about the children's eye health. Parents were queried on several key aspects: they were asked whether their children had ever been diagnosed with specific ocular conditions, such as squint (strabismus) or amblyopia (lazy eye). Additionally, the questionnaire sought information regarding any observable signs of poor eyesight in their children, including behaviours like squinting, sitting unusually close to the television, or frequent eye rubbing. Furthermore, to understand the continuity and extent of prior medical attention, parents were asked if their child had undergone any eye examinations, and if so, the time of these examinations. The chi-squared-test was used in statistical analysis of the questionnaire information (squint, amblyopia, bed eyesight, prior examinations).

TOSTs were performed between results obtained at schools and in the clinic with equivalence limits in the range − 0.1 to 0.1 and a significance level of α = 0.05. In accordance with the study's rigorous data integrity standards, any participant for whom there was incomplete or missing information in the research protocol was excluded from the statistical analysis.

The images of the fundus were analysed using a CNN model specifically designed for this research for the automatic detection of diseases such as glaucoma, diabetic macular oedema, and macular degeneration. The architecture of the CNN began with a convolution layer followed by a batch normalization layer; its function was to standardize the inputs across each layer, thus effectively enhancing the model's training efficiency and augmenting its stability^29^. Next, a rectified linear unit (ReLU) activation function was applied^30^.

Subsequently, a second convolution layer followed by a batch normalization layer was employed, sharing the same aim as the first, namely, facilitating stabilization of the learning process and expediting the training phase^29^. An activation layer ensued, deploying the ReLU function to inject a nonlinear characteristic into the model^31,32^. This stage was succeeded by a dropout layer strategically designed to randomly nullify a fraction of the input units throughout the training process as a countermeasure to overfitting^30^. The architecture continued with a second dense layer, whose purpose was to reduce the input's dimensionality, thereby refining the network structure.

**Figure 1:**
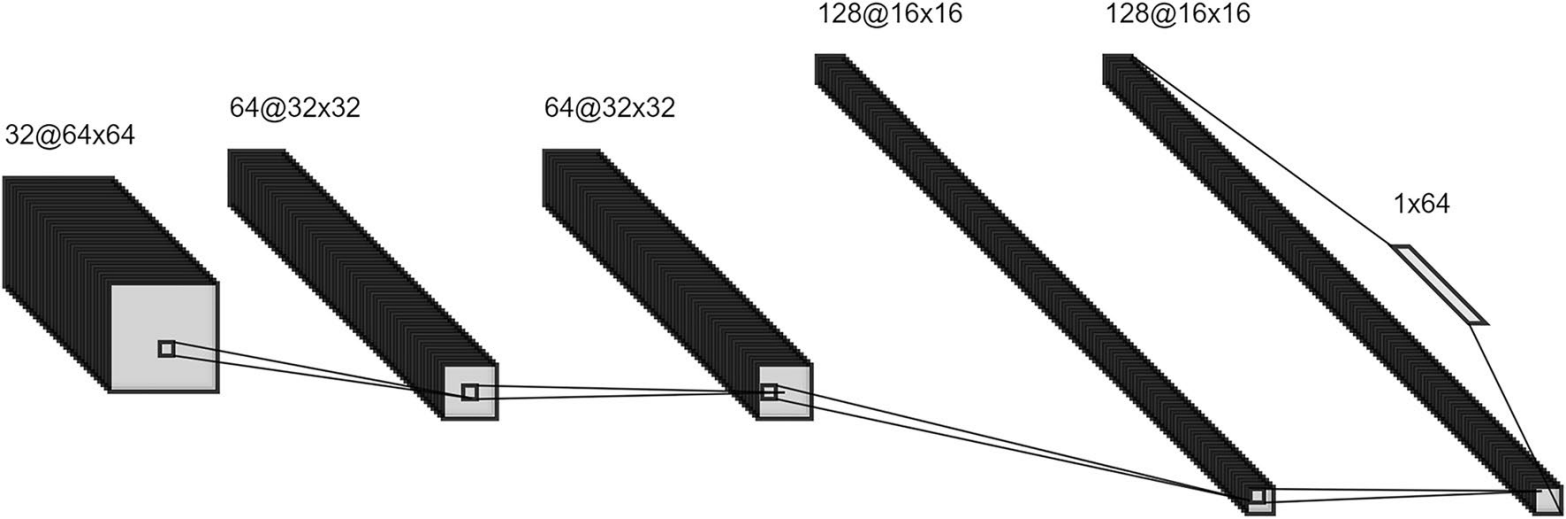
Architecture of the convolutional neural network for automatic diagnosis of retinal pathologies.

Furthering the complexity of the network, a third convolution layer followed by a batch normalization layer and a second activation layer (ReLU) were incorporated. The network then introduced a second dropout layer, sharing the objective of the first, primarily to mitigate 4269 layer, with each neuron representing the classes that the model was trained to predict. The entire sequence was then passed through a sigmoid function, providing a probabilistic interpretation of the model's outcomes^33^ (Fig. 1).

The CNN model was trained on the publicly available ODIR dataset, which contains 5000 images, was equipped to diagnose various pathologies, including drusen, glaucoma, and diabetic macular oedema^25,34^. This dataset was partitioned into training (70%), validation (20%), and test (10%) subsets. The rationale behind this split is to ensure that the model learns from the most substantial portion of the data (training set) and then uses the validation set to fine-tune its parameters and prevent overfitting, while the test set is utilized to evaluate the model's performance on unseen data, offering a realistic measure of its predictive capabilities^35^.

During each epoch of the training process, the original dataset underwent dynamic augmentation through the application of a series of stochastic transformations. This procedure included the rotation of images across a spectrum of angles, varying from minimal to considerable degrees. In addition to rotations, image distortions were also introduced, like scale, shifts in the image position (both horizontally and vertically), shearing, and zooming. By applying these distortions, the model was trained to identify and understand the relevant features of an image even when they were presented in varied proportions or perspectives. The augmentation process was performed on-the-fly during the training. This means that in each epoch, the images were transformed in a new and randomized manner, significantly expanding the diversity of the training data without the need for additional data collection. This approach not only prevented the model from overfitting to the training data but also enhanced its ability to generalize to new, unseen data, a crucial factor in the successful deployment of AI models in practical applications.

During the training process with the ODIR dataset^21,25,34,36^, the model accuracy was computed (the ratio of correctly predicted observations to the total observations) on both the validation and test sets, yielding a value of 89.3%. Furthermore, to comprehensively evaluate the performance of the CNN model in the automatic detection of diseases such as glaucoma, diabetic macular oedema, and macular degeneration in children, its predictions were compared with diagnoses made by ophthalmologists using the Dice coefficient.

The study received approval from the regional research ethics board, specifically the Bioethics Committee at Medical University in Lublin, with reference number KM-0254/185/2021 on June 24, 2021. All methods were implemented in adherence to the relevant guidelines and regulations and were in compliance with the Declaration of Helsinki. Prior to the commencement of the study, informed consent was obtained from the parents of the participating children. The consent form provided detailed information about the study protocol. Ophthalmological examinations were performed only on children whose parents had provided signed consent for their participation in the study.

The research project entitled "Pilot Program for Myopia Prevention in Children from Grades 1–3 in the Lubelskie Voivodeship" was implemented based on a granted targeted subsidy, under Agreement No. MZ/UML/2021/1206/645 dated August 6, 2021, and Annex No. 1 dated December 29, 2021, and Annex No. 2 dated May 9, 2022, concluded between the State Treasury-Minister of Health and the Medical University of Lublin.

## Data Availability

The data that support the findings of this study are available from the corresponding author upon reasonable request.
